# Dynamics of chromatin accessibility during TGF-**β-**induced EMT of Ras-transformed mammary gland epithelial cells

**DOI:** 10.1038/s41598-017-00973-4

**Published:** 2017-04-26

**Authors:** Mayu Arase, Yusuke Tamura, Natsumi Kawasaki, Kazunobu Isogaya, Ryo Nakaki, Anna Mizutani, Shuichi Tsutsumi, Hiroyuki Aburatani, Kohei Miyazono, Daizo Koinuma

**Affiliations:** 1 Department of Molecular Pathology, Graduate School of Medicine, The University of Tokyo, Bunkyo-ku, Tokyo, 113-0033 Japan; 2 Genome Science Division, Research Center for Advanced Science and Technology (RCAST), The University of Tokyo, Meguro-ku, Tokyo, 153-8904 Japan; 3 Laboratory of Human Carcinogenesis, Center for Cancer Research, National Cancer Institute, Bethesda, MD 20892 USA; 4 Division of Molecular Biotherapy, Cancer Chemotherapy Center, Japanese Foundation for Cancer Research, Ariake, Koto-ku, Tokyo, 135-8550 Japan

## Abstract

Epithelial-mesenchymal transition (EMT) is induced by transforming growth factor (TGF)-β and facilitates tumor progression. We here performed global mapping of accessible chromatin in the mouse mammary gland epithelial EpH4 cell line and its Ras-transformed derivative (EpRas) using formaldehyde-assisted isolation of regulatory element (FAIRE)-sequencing. TGF-β and Ras altered chromatin accessibility either cooperatively or independently, and AP1, ETS, and RUNX binding motifs were enriched in the accessible chromatin regions of EpH4 and EpRas cells. Etv4, an ETS family oncogenic transcription factor, was strongly expressed and bound to more than one-third of the accessible chromatin regions in EpRas cells treated with TGF-β. While knockdown of Etv4 and another ETS family member Etv5 showed limited effects on the decrease in the E-cadherin abundance and stress fiber formation by TGF-β, gene ontology analysis showed that genes encoding extracellular proteins were most strongly down-regulated by Etv4 and Etv5 siRNAs. Accordingly, TGF-β-induced expression of Mmp13 and cell invasiveness were suppressed by Etv4 and Etv5 siRNAs, which were accompanied by the reduced chromatin accessibility at an enhancer region of *Mmp13* gene. These findings suggest a mechanism of transcriptional regulation during Ras- and TGF-β-induced EMT that involves alterations of accessible chromatin, which are partly regulated by Etv4 and Etv5.

## Introduction

Transforming growth factor (TGF)-β is the prototype of the TGF-β family proteins. TGF-β regulates various cellular responses, e.g. cytostasis, cell differentiation, apoptosis, cell motility, and extracellular matrix production^1^; in addition, disruption of TGF-β signaling is related to various diseases^2, 3^. Smad family proteins transduce intracellular TGF-β signaling from cell membrane to the nucleus^4–6^. In the nucleus, Smad proteins cooperate with various transcription factors, transcriptional coactivators and corepressors, and regulate transcription of target genes^7–9^.

TGF-β plays bi-directional roles in the progression of cancer^10^. In the early tumor stages, TGF-β behaves as a tumor suppressor by inhibiting proliferation of epithelial cells through regulation of the expression of c-Myc and cyclin-dependent kinase inhibitors, and by inducing apoptosis^11, 12^. In the later stage of cancer, TGF-β acts as a tumor promoter^13^, and recent findings have revealed that epithelial-mesenchymal transition (EMT) plays important roles in this process^14, 15^.

The EMT is a crucial step in which epithelial cells functionally and morphologically differentiate into mesenchymal cells, and this is important in the process of embryonic development and wound healing^16^. It has also been reported that EMT contributes to the tumor progression^17, 18^. In the process of EMT, cancer cells lose tight cell-cell junctions and acquire mesenchymal phenotypes. Consequently, they invade surrounding blood vessels and lymph vessels, and disseminate to distant tissues and organs^19^. The EMT is accompanied by reduced expression of epithelial markers, including E-cadherin and epithelial splicing regulatory protein 2 (ESRP2)^20^, and upregulation of the expression of mesenchymal markers, including N-cadherin, fibronectin, and α-smooth muscle actin (α-SMA). Cells become spindle-shaped and motile with actin stress fiber formation. At the adherens junctions, E-cadherin plays important roles in cell-cell attachment of epithelial cells. The intracellular domain of E-cadherin binds cortical actin through α-catenin and β-catenin, and loss of E-cadherin is essential for EMT. Several extracellular stimuli induce EMT, and previous studies have revealed that induction of EMT by TGF-β requires Ras signaling^21–23^. Indeed, MDCK cells and EpH4 cells, frequently used for analyses of EMT, cause EMT only when Ras signaling is activated^24^.

EMT is a process of trans-differentiation of epithelial cells which involves dynamic changes in DNA methylation and histone tail modifications^25^, and chromatin accessibility of DNA binding factors is determined as a result of such complex epigenetic modifications. In the present study, we performed global mapping of the accessible chromatin regions in mouse mammary gland epithelial EpH4 cells and their H-Ras-transformed derivative, EpRas cells, using formaldehyde-assisted isolation of regulatory element (FAIRE)-sequencing (seq). This allowed us to analyze the mechanisms of transcriptional regulation during TGF-β-induced EMT. We found that EMT is regulated through alteration of chromatin accessibility by Ras-induced transformation and TGF-β signaling, and identified an enrichment of AP1, ETS, and RUNX-like binding motifs in the FAIRE-positive, accessible chromatin regions in both EpH4 and EpRas cells. We found up-regulation of the oncogenic ETS transcription factors Etv4 (also known as Pea3 or E1af) and Etv5 (also known as Erm) in EpRas cells. While knockdown of Etv4 and Etv5 (Etv4/5) only minimally affected the decrease in E-cadherin protein expression by TGF-β, comprehensive analysis of target genes of Etv4 and Etv5 revealed their potential role in expression of extracellular proteins. FAIRE-seq after knockdown of Etv4/5 also showed an inverse correlation with the effect of TGF-β on chromatin accessibility at a genome-wide level. Accordingly, knockdown of Etv4/5 in EpRas cells reduced the chromatin accessibility at the *Mmp13* gene locus and cell invasiveness. These findings suggest a mechanism of EMT-related transcriptional regulation involving the chromatin accessibility that is partly regulated by Etv4 and Etv5 in cancer cells.

## Results

### Regulation of accessible chromatin regions by TGF-β and Ras signaling in mouse mammary gland epithelial cells

**Figure 1:**
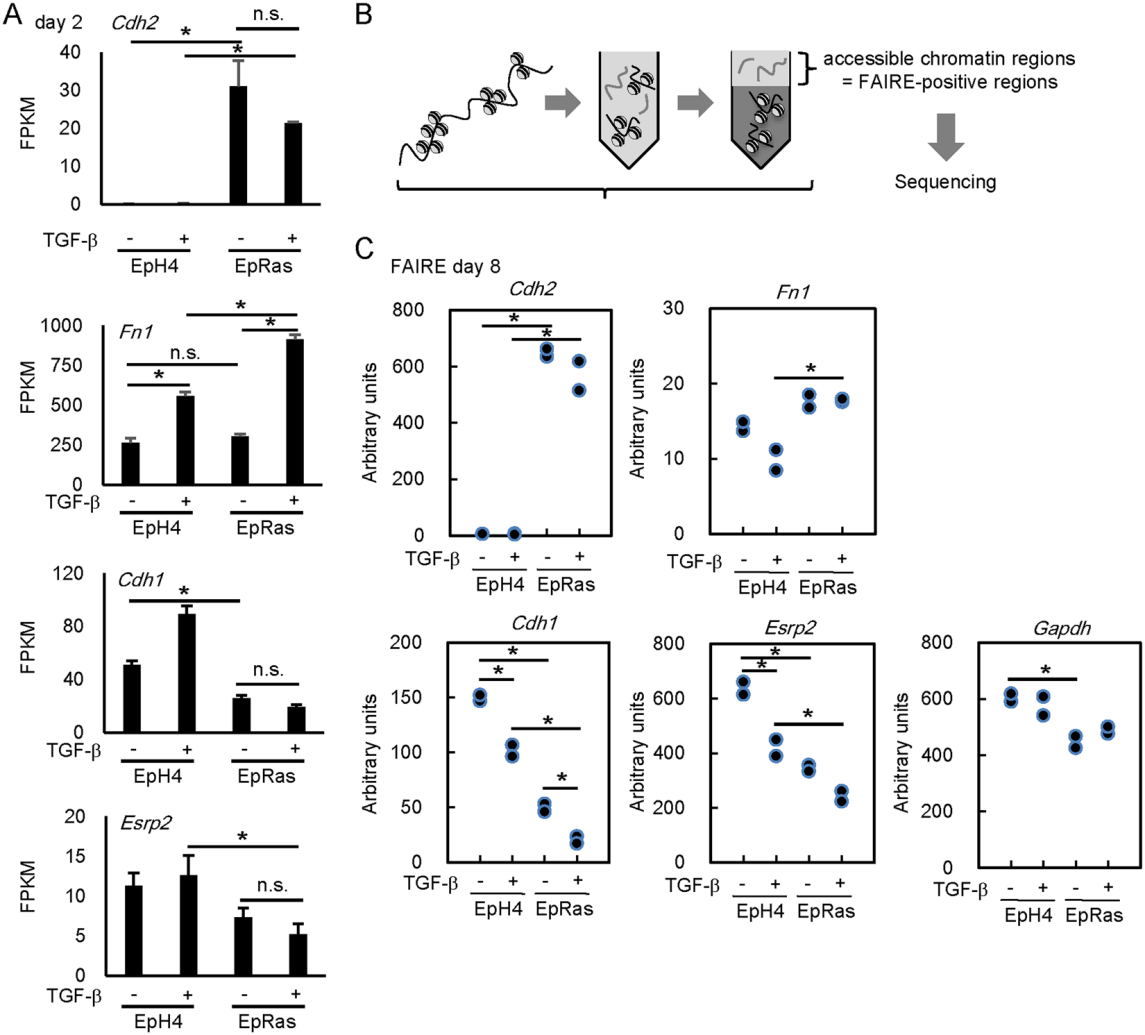
Regulation of accessible chromatin regions (FAIRE-positive regions) by TGF-β at EMT-related gene loci in EpH4 and EpRas cells. (**A**) Expression levels of *Cdh2* (encoding N-cadherin), *Fn1* (encoding fibronectin), *Cdh1* (encoding E-cadherin), and *Esrp2* mRNA evaluated by RNA-seq. Error bars represent 95% confidence interval (CI). EpH4 and EpRas cells were stimulated with TGF-β for 48 h, and expression levels of each mRNA were examined. FPKM, fragments per kilobase of exon per million mapped sequence reads. *p < 0.05, n.s.: not significant. (**B**) Schematic representation of FAIRE-seq. Cells were treated with 1 ng/ml TGF-β, fixed with formaldehyde, sheared by sonication, and phenol/chloroform extracted. (**C**) After treatment with 1 ng/ml TGF-β for 8 days, chromatin accessibility at the promoter regions of *Cdh2*, *Fn1*, *Cdh1, Esrp2*, and *Gapdh* loci was examined in EpH4 and EpRas cells by FAIRE-qPCR analysis. The experiment was performed in two biological replicates. Representative data of the two independent experiments are shown. *p < 0.05.

The TGF-β-induced EMT is accompanied by upregulation of mesenchymal markers and downregulation of epithelial markers. It has been reported that TGF-β induces the EMT of EpRas cells (EpH4 cells transformed with H-RasG12V), but not of their parental cells^24^. We examined the gene expression profiles of these cell lines by RNA-seq after stimulation with TGF-β for 48 h. We found that the expression of mesenchymal marker *Cdh2* (encoding N-cadherin) was increased in EpRas cells regardless of the stimulation with TGF-β. In contrast, treatment with TGF-β potently induced the expression of *Fn1* (encoding fibronectin) at the mRNA level both in EpRas cells and in EpH4 cells. The expression of epithelial gene *Cdh1* (encoding E-cadherin) was decreased in the EpRas cells compared to EpH4 cells (Fig. 1A). Although the expression levels of *Cdh1* and another epithelial gene, *Esrp2*, were not significantly decreased by 48-h treatment with TGF-β in EpRas cells, and the expression level of *Cdh1* was even increased by TGF-β in EpH4 cells, we found enhanced down-regulation of *Cdh1* and *Esrp2* by prolonged culture of EpRas cells with TGF-β (8 days, Supplementary Fig. S1), suggesting continued alteration of transcriptional program by long-term stimulation, as previously described^26^.

To examine the epigenetic status of chromatin in the gene loci that encode EMT-related factors in these cells, we performed FAIRE quantitative PCR (FAIRE-qPCR) at day 8. FAIRE-qPCR determines the accessible chromatin regions for DNA binding factors (FAIRE-positive regions), which are associated with transcriptional regulatory activity (Fig. 1B). We found that chromatin accessibility at the promoter of the *Cdh2* gene was increased in EpRas cells regardless of TGF-β stimulation. In contrast, high chromatin accessibility was observed in both EpH4 and EpRas cells at the promoter of the *Fn1* gene, another well-known mesenchymal marker (Fig. 1C). Furthermore, we found that chromatin accessibility was reduced in EpRas cells at the epithelial marker genes *Cdh1* and *Esrp2* compared to *Gapdh*, and this reduction was enhanced by TGF-β stimulation. These findings suggest that the varied regulation of chromatin accessibility is related to expression of certain genes during EMT.

### Genome-wide profiling of chromatin accessibility by FAIRE-seq during EMT

**Figure 2:**
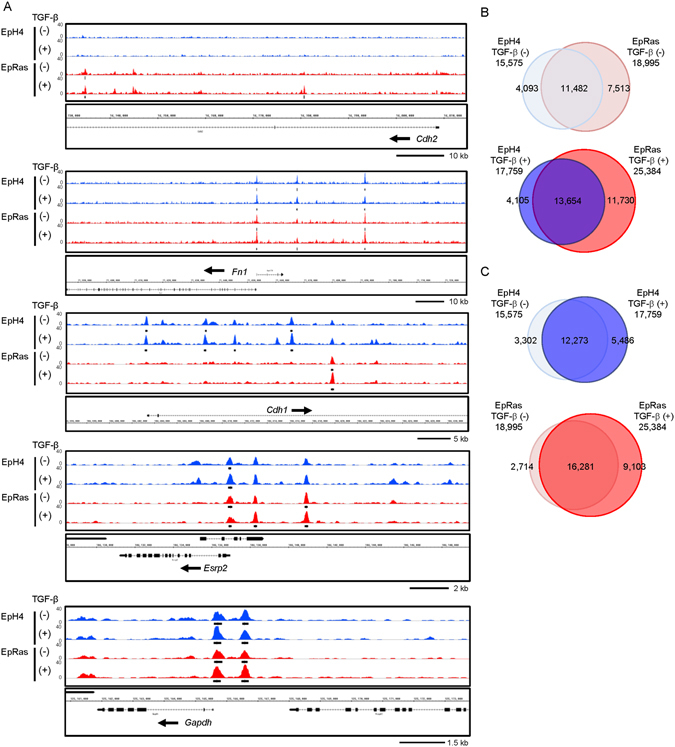
Genome-wide profiling of accessible chromatin regions by FAIRE-seq in EpH4 and EpRas cells. (**A**) Accessible chromatin regions at the *Cdh2*, *Fn1*, *Cdh1*, *Esrp2*, and *Gapdh* loci, determined by FAIRE-seq. The EpH4 and EpRas cells were treated with 1 ng/ml TGF-β for 8 days. Each graph track represents the combined data obtained from two biological replicates. The vertical axis represents read tag densities and the scales are set to the same value. The arrows next to the gene symbols represent the direction of transcription. The black bars show significant FAIRE-positive regions with q < 1.0 × 10^−4^. (**B**,**C**) Venn diagrams comparing the FAIRE-positive regions (q < 1.0 × 10^−4^) among EpH4 and EpRas cells treated with or without 1 ng/ml TGF-β for 8 days.

We next performed deep sequencing of FAIRE samples to determine how TGF-β and Ras transformation regulate the chromatin accessibility and expression of EMT-related factors during the process of EMT. Statistically significant accessible chromatin regions (FAIRE peaks) were determined using an established peak calling program^27^. We found that statistically significant FAIRE peaks located within the *Cdh2* gene were found in EpRas cells but not in EpH4 cells (Fig. 2A, top panel, black bars). The FAIRE peaks upstream of the promoter of the *Fn1* gene were present in both EpH4 and EpRas cells (second panel). Many of the FAIRE peak signals in *Cdh1* were decreased in EpRas cells (third panel). These results were largely in agreement with those obtained by FAIRE-qPCR (Fig. 1C), except for the *Esrp2* gene locus (fourth panel). We then determined condition-specific accessible chromatin regions by analyzing the overlap of significant FAIRE regions between the samples, and found that in the absence of TGF-β, 26.3% of the accessible chromatin regions in EpH4 cells (4,093 regions) were unique for these cells, and not shared with EpRas cells (Fig. 2B, upper panel). In EpRas cells treated with TGF-β for 8 days, 25,384 accessible chromatin regions were observed, and 46.2% of them were unique for these cells, and not shared with EpH4 cells (Fig. 2B, bottom panel). The effect of TGF-β was further evaluated by comparing the number of accessible chromatin regions in each cell line (Fig. 2C). We found increased accessible chromatin regions by TGF-β treatment in EpRas cells (9,103 regions), while a limited number of the regions (2,714 regions) were lost after TGF-β stimulation.

### Enrichment of AP1, ETS, and RUNX binding motifs in the accessible chromatin regions both in EpH4 and EpRas cells

**Figure 3:**
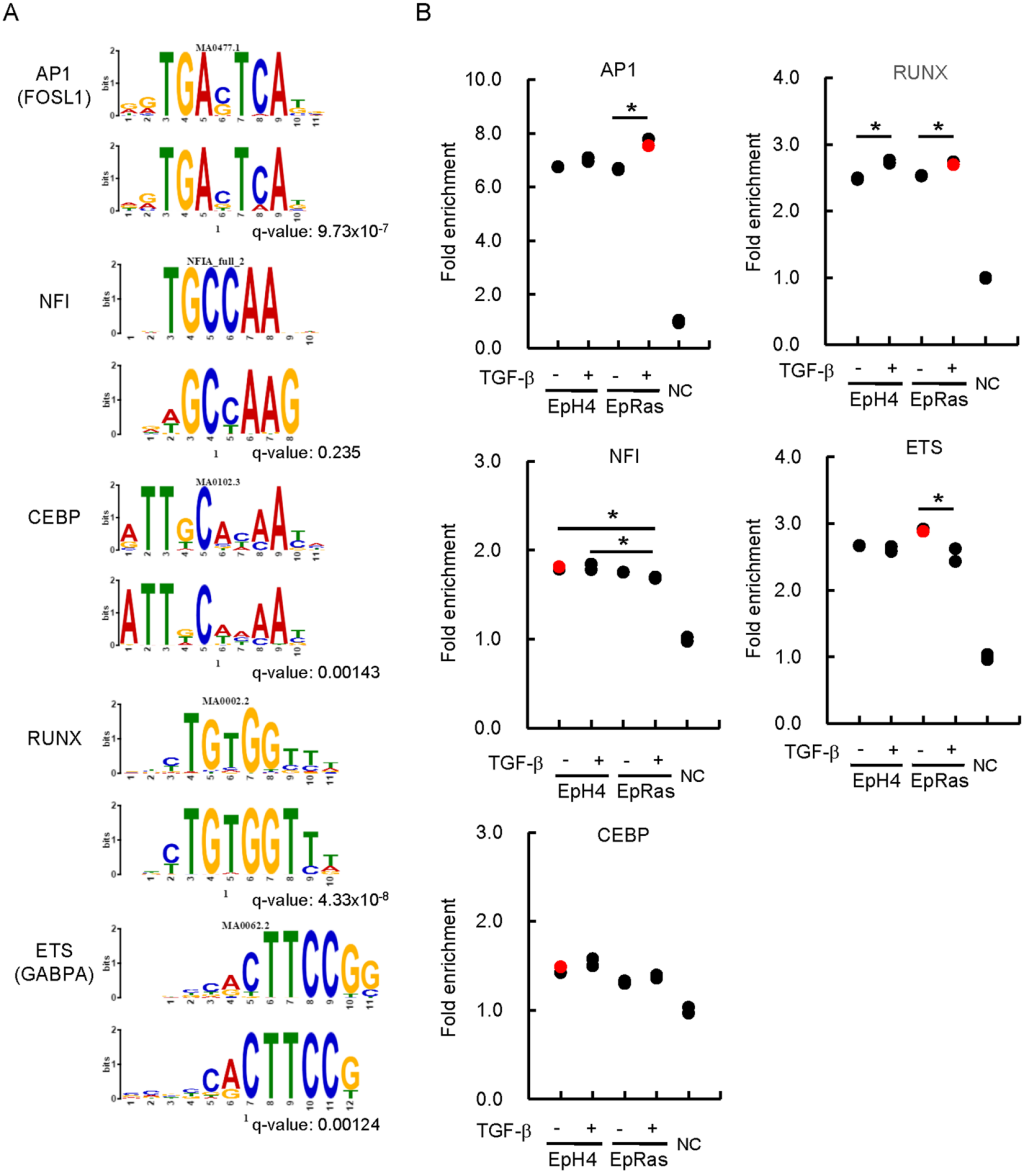
Motif enrichment analysis of the context-specific FAIRE-positive regions of EpH4 and EpRas cells. (**A**) *De novo* motif prediction of accessible chromatin regions. After exclusion of promoter regions and the sequence repeat areas, 500 bp sequences flanking the middle position of each FAIRE peak region were analyzed using the Gibbs motif sampler function of CisGenome. The following parameters were used: K = 15, mean length lambda = 10 bp, and maximal length = 20 bp. Alignments to the known transcription factor binding motifs are shown as sequence logos (upper panel for each motif, most similar known motifs identified by TOMTOM; lower panel for each motif, motifs calculated from FAIRE-positive regions). q value (minimal false discovery rate required to include the motif) is shown for each motif. (**B**) Fold enrichment values of the identified motifs were calculated compared to two sets of matched control genomic regions picked up by Gibbs motif sampler (NC). Red dots show the datasets from which the motif was identified in (**A**). *p < 0.05. Note that the enrichment of every motif was significantly higher than the control genomic regions in all four samples, and the results of statistical analyses were omitted for simplicity.

We performed transcription factor binding motif prediction and enrichment analyses of accessible chromatin regions in EpH4 and EpRas cells treated with and without TGF-β. Repeat sequences and transcriptional starting sites were excluded from the calculation, because condition-specific accessible chromatin regions were mostly observed in enhancer regions^28^. We identified an AP-1-like binding motif (shown as a FOSL1 motif in Fig. 3A, first panel; in Fig. 3B, red dots show the datasets from which the motif was calculated) from all the data evaluated, i.e. both from EpH4 and EpRas cells with or without TGF-β treatment. We also identified NF1-like and CEBP-like binding motifs from the FAIRE-seq data of EpH4 cells in the absence of TGF-β (Fig. 3A, second and third panels). We also identified a RUNX-like binding motif from the data of TGF-β-treated EpRas cells (Fig. 3A, fourth panel). An ETS-like binding motif (shown as GABPA in Fig. 3A, bottom panel) was identified from the data of untreated EpRas cells.

We then calculated the relative enrichment of those motifs in each FAIRE-seq dataset compared to the control genomic regions (Fig. 3B). Frequencies of those motifs in the FAIRE-positive regions in all four conditions (EpH4 and EpRas cells, with and without TGF-β) were significantly higher than the control genomic regions (shown as NC). However, there were significant but limited extents of differences in the enrichment of those motifs between the cell lines and/or TGF-β treatment.

We therefore focused on the differential expression of transcription factors that bind to those motifs. In general, we did not find significant differences in the expression of most of the transcription factors that bind to AP-1 (Supplementary Fig. S2A), NF1, CEBP, and RUNX motifs (Supplementary Fig. S2B) between EpH4 and EpRas cells. The expression levels of the ETS family genes Elk1, Ets2, Etv3, and Etv6 were down-regulated in EpRas cells compared to EpH4 cells in the presence of TGF-β (Supplementary Fig. S2C), while we found up-regulation of Etv4 and Etv5 in EpRas cells both in the TGF-β treated and untreated conditions.

### Identification of Etv4 and Etv5 as up-regulated ETS family transcription factors in EpRas cells

**Figure 4:**
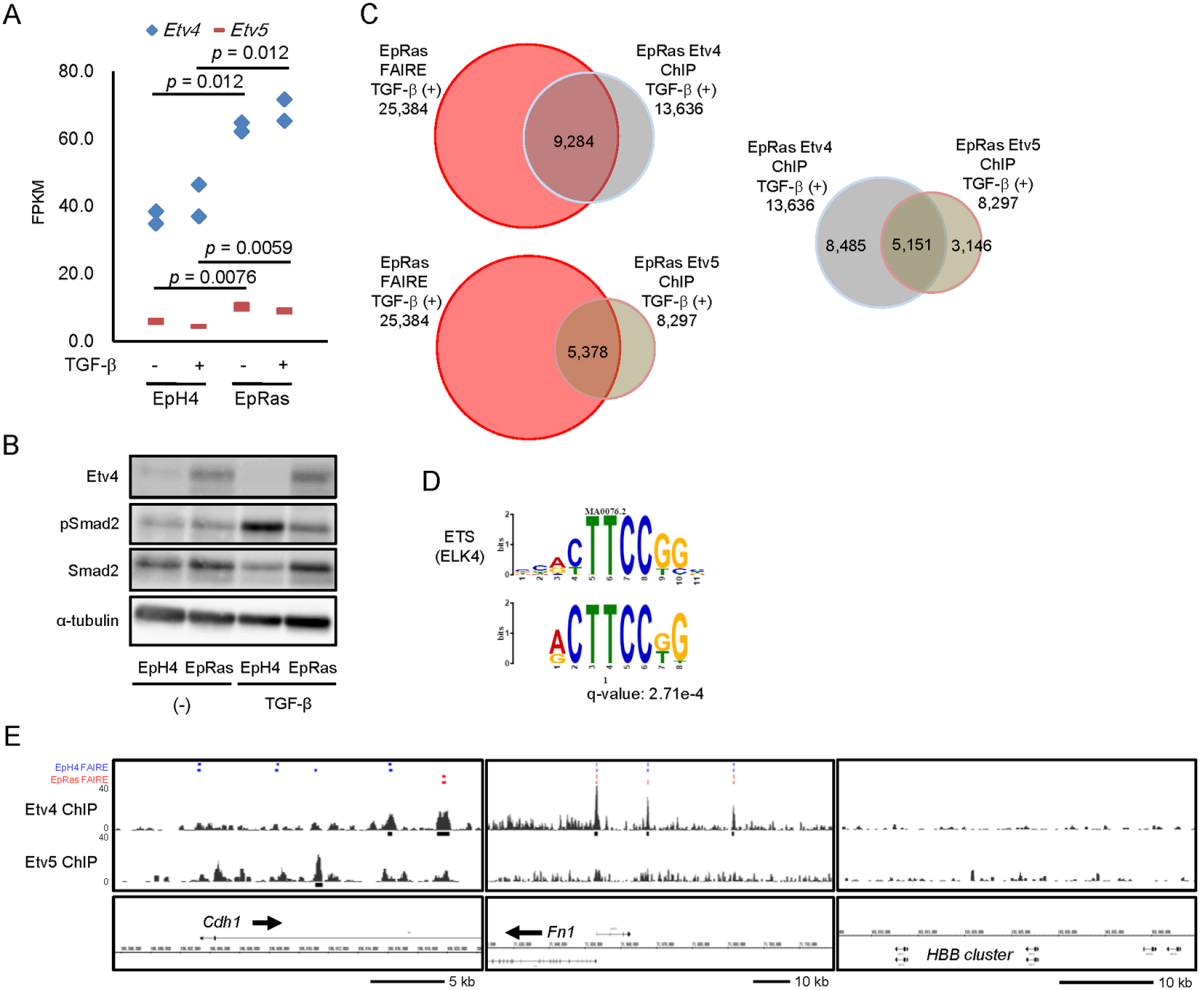
Global analysis of Etv4 and Etv5 binding regions in the genome. (**A**) Expression of Etv4 and Etv5 by RNA-seq of EpH4 and EpRas cells treated with 1 ng/ml TGF-β for 48 h. FPKM, fragments per kilobase of exons per million mapped fragments. The experiment was performed in two biological replicates. Data are a subset of the findings shown in Supplementary Fig. S2C. (**B**) Immunoblot analysis of endogenous Etv4 protein expression in EpH4 and EpRas cells. pSmad2, phosphorylated Smad2. (**C**) Relationship between the anti-Etv4 and anti-Etv5 ChIP-seq data and FAIRE-seq data obtained from TGF-β-treated EpRas cells. (**D**) *De novo* motif prediction identifies ETS family (ELK4) binding motif, and shown as in Fig. 3A. Anti-Etv4 ChIP-seq data were used for enriched motif calculation. q value (minimal false discovery rate required to include the motif) is shown. (**E**) Etv4 and Etv5 binding regions at the *Cdh1* and *Fn1* gene loci. *HBB* cluster region is shown as a control. Black bars represent the significant Etv4 and Etv5 binding regions. Significant FAIRE-positive regions in EpH4 and EpRas cells, without (first row) and with (second row) TGF-β stimulation, are shown as blue and red bars, respectively, as a reference (shown in Fig. 2A).

We focused on the expression of Etv4 and Etv5, both are known as oncogenic ETS family transcription factors. Etv4 was one of the highly expressed ETS family transcription factors, and its expression was increased by Ras transformation both at mRNA (Fig. 4A: an enlarged data extracted from Supplementary Fig. S2C) and protein levels (Fig. 4B). The Ras transformation also increased the expression of Etv5, an ETS family member functionally related with Etv4 (Fig. 4A).

We then performed ChIP-seq analyses using the antibodies to Etv4 and Etv5 (Fig. 4C). We found that as many as 9,284 (36.6%) and 5,378 (21.2%) accessible chromatin regions in the EpRas cells stimulated with TGF-β for 8 days were bound by Etv4 and Etv5, respectively, at the point of 1.5 h after TGF-β stimulation (Fig. 4C, left panels). Overlap between the Etv4 and Etv5 binding was observed at 5,151 regions, suggesting some differences in the binding specificity between the two ETS family factors. Motif calculation of the Etv4 ChIP-seq data identified ETS binding motif as one of the most enriched elements, which was shared by the Etv5 ChIP-seq data at a comparable frequency (Supplementary Fig. S3A), suggesting that the acquired data are valid (Fig. 4D). Based on the peak calling and target annotation results, we found that Etv4 and Etv5 bound to the *Cdh1* and *Fn1* loci but not in the *HBB* cluster, which served as a control (Fig. 4E).

### Involvement of Etv4 and Etv5 in the cell invasiveness regulated by TGF-β

**Figure 5:**
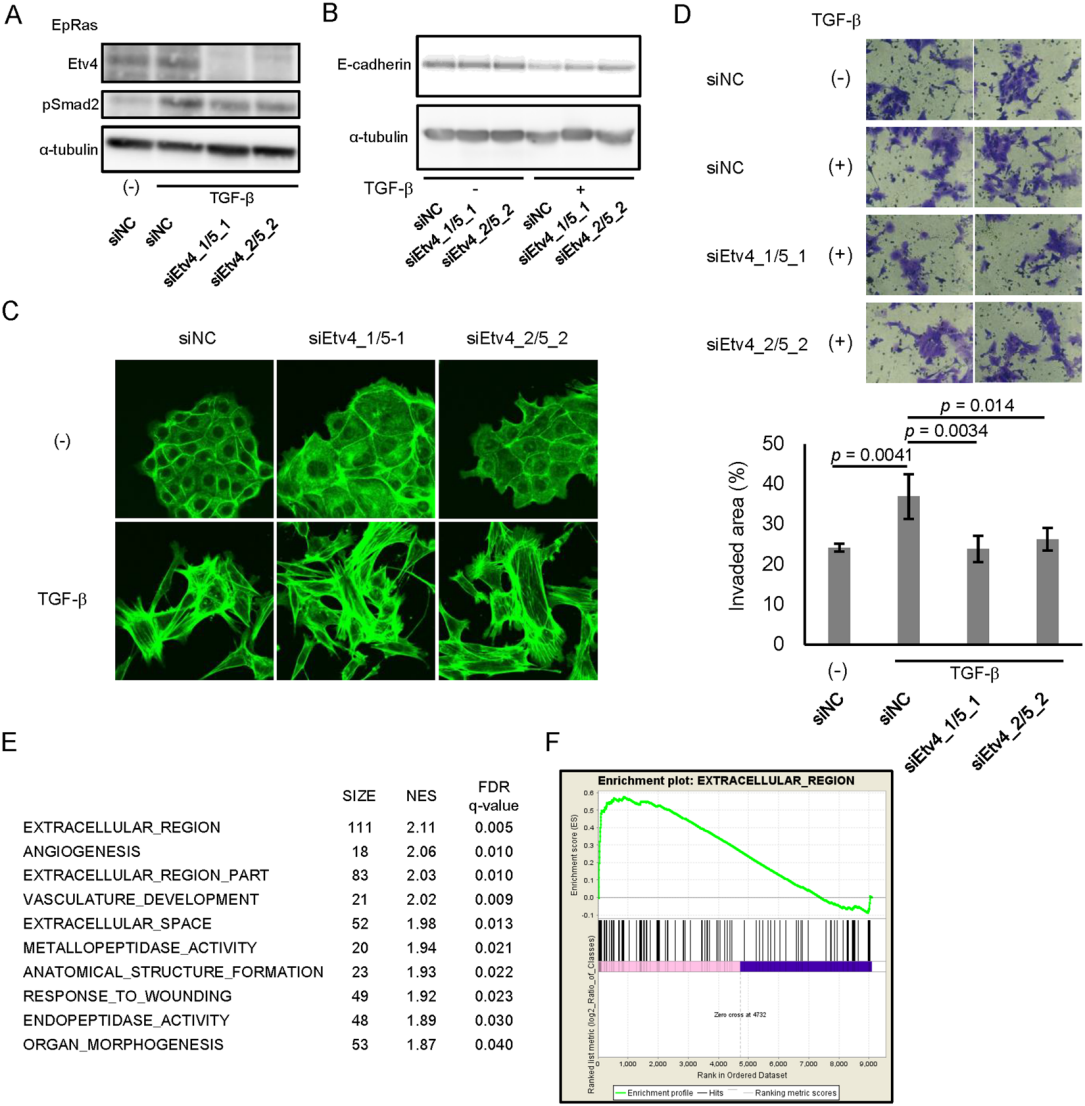
Effect of Etv4 and Etv5 siRNAs on stress fiber formation, cell invasiveness and global gene expression in EpRas cells. (**A**) Effect of the siRNAs on the expression of Etv4 protein. EpRas cells were transfected with the siRNAs as indicated. After 24 h of incubation, cells were treated with TGF-β for additional 48 h and lysed for immunoblotting analysis. (**B**) Effect of Etv4/5 siRNAs on the TGF-β-induced down-regulation of E-cadherin protein. siRNA-transfected cells were treated with TGF-β for 8 days. Transfection of siRNAs were repeated at day 2 and 5. (**C**) Effect of Etv4/5 siRNAs on the stress fiber formation induced by TGF-β. F-actin formation of EpRas cells was evaluated by phalloidin staining. Cells were transfected with siRNAs for Etv4/5 and stimulated with TGF-β for 48 h. (**D**) Matrigel invasion assay in EpRas cells. Cells were transfected with the indicated siRNAs and then seeded on the Matrigel-coated plate, incubated for 48 h with TGF-β, and fixed. (upper panels) Representative images of the cells that migrated through the Matrigel-coated membrane. (bottom graph) Quantified data representing the means of four independent experiments. Error bars, standard deviations. (**E** and **F**) EpRas cells were transfected with siRNAs for Etv4/5 and treated with 1 ng/ml TGF-β for 48 h. Then, RNA-seq and ontology analysis using GSEA were performed. Genes with maximum FPKM (fragments per kilobase of exon per million mapped sequence reads) values ≥5 among the samples were selected for evaluation. (**E**) A list of top-enriched c5 (gene ontology) gene sets (MSigDB) downregulated by siEtv4/5 in the absence of TGF-β. The averaged gene expression data from the two distinct sets of siEtv4/5-transfected cells were compared to the data from the siNC-transfected cells. SIZE: number of genes in the phenotype, NES: normalized enrichment score, FDR: false discovery rate﻿. (**F**) An enrichment plot showing the up-regulation of a gene set “EXTRACELLULAR REGION” by Etv4 and Etv5, which was identified as the top-enriched phenotype by GSEA.

We then knocked down the expression of Etv4 by two different small interfering RNAs (siRNAs; siEtv4_1 and siEtv4_2) (Fig. 5A). We also knocked down the expression of Etv5 by siRNAs (siEtv5_1 and siEtv5_2) simultaneously to address known functional redundancy of Etv4 and Etv5 genes in EpRas cells. We found that knockdown of both Etv4/5 by their siRNAs only minimally attenuated the downregulation of E-cadherin protein after 8 days of TGF-β stimulation in EpRas cells (Fig. 5B). These findings suggest that, although Etv4 and Etv5 bind to the *Cdh1* gene locus, they are not essential factors of Cdh1 suppression during TGF-β-induced EMT. We also evaluated the effect of Etv4/5 siRNAs on TGF-β-induced stress fiber formation. As shown in Fig. 5C, loss of Etv4/5 expression did not attenuate the TGF-β-induced morphological change and stress fiber formation in EpRas cells (﻿Fig. 5C). In contrast, we found that TGF-β-induced cell invasiveness was suppressed by siRNAs for Etv4/5 (Fig. 5D). These data suggested that Etv4 and Etv5 regulate a subset of EMT-related changes in the gene expression and cellular phenotype.

To determine the roles of Etv4 and Etv5 at a genome-wide level, we performed RNA-seq analysis using EpRas cells transfected with two distinct pairs of Etv4/5 siRNAs and stimulated the cells with TGF-β for 48 h. By combining the ChIP-seq and RNA-seq data, the Etv4/5–regulated genes were listed in Supplementary Table S1. The Gene Set Enrichment Analysis (GSEA) using the gene expression data identified several enriched gene ontology gene sets (Fig. 5E). We found that some of the top enriched phenotypes regulated by Etv4 and Etv5 in EpRas cells were related to upregulation of the genes encoding extracellular proteins both in the unstimulated (Fig. 5F) and TGF-β-stimulated (Supplementary Fig. S3B) conditions, suggesting Etv4 and Etv5 may promote secretion of certain extracellular proteins related to pro-tumorigenic functions.

### Regulation of Mmp13 expression by Etv4 and Etv5 through the alteration of chromatin accessibility

**Figure 6:**
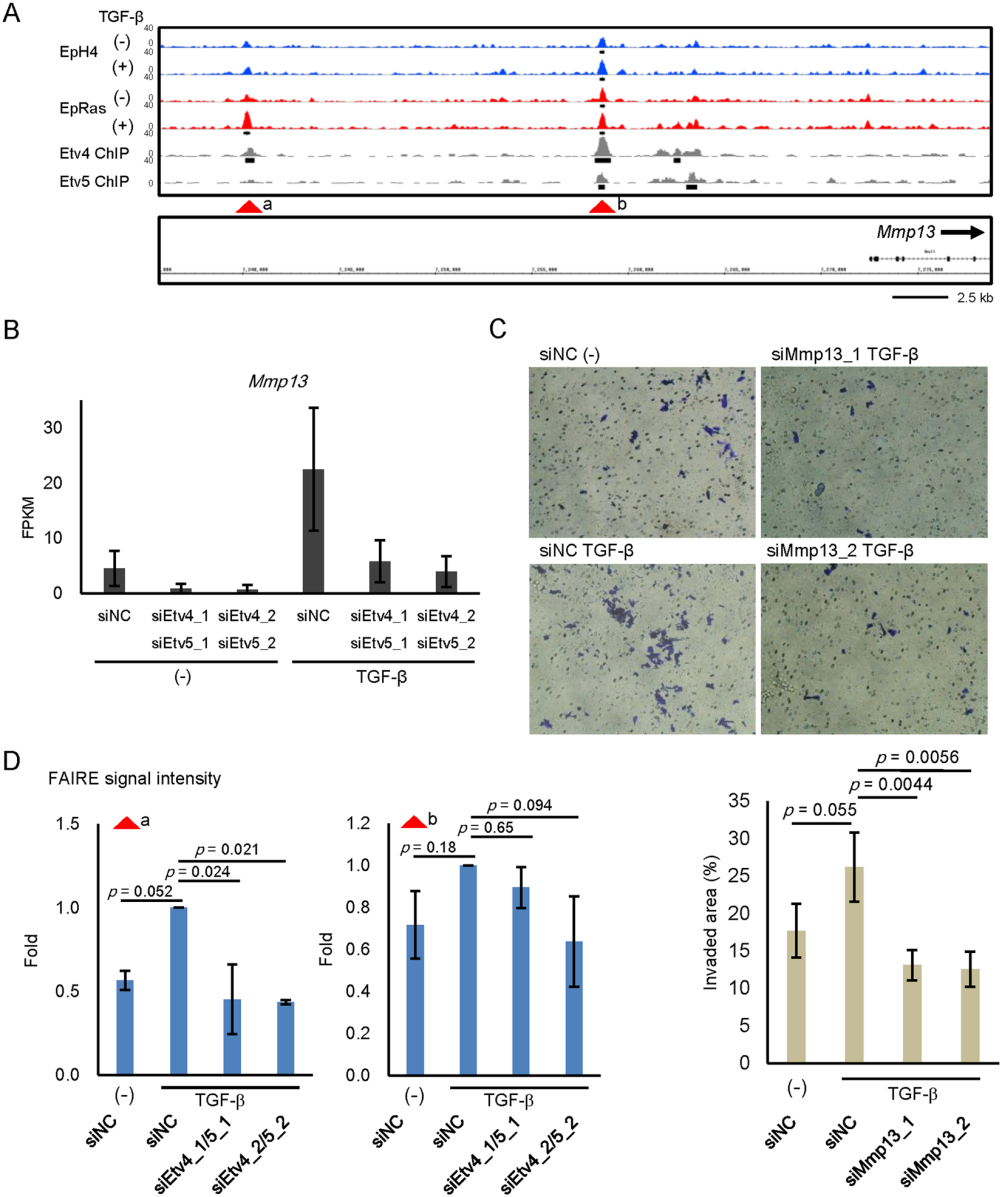
Regulation of Mmp13 expression by Etv4 and Etv5. (**A**) FAIRE-seq and ChIP-seq data at the matrix metalloproteinase 13 (*Mmp13*) gene locus as a target of Etv4 and 5. Data are shown as in Figs 2A and 4E. Arrow heads show the positions evaluated by FAIRE-qPCR in (**D**). (**B**) RNA-seq data of siEtv4/5-transfected EpRas cells treated with TGF-β. siNC; negative control siRNA. Error bars, 95% confidence intervals. (**C**) Matrigel invasion assay in EpRas cells. Cells were transfected with the indicated siRNAs and then seeded on the Matrigel-coated plate, incubated for 48 h with TGF-β, and fixed. (upper panels) Representative images of the cells that migrated through the Matrigel-coated membrane. (bottom graph) Quantified data representing the means of four independent experiments. Error bars, standard deviations. (**D**) Changes in the FAIRE-seq signal intensities by Etv4/5 siRNAs at FAIRE-positive regions of *Mmp13* gene locus shown in (**A**). EpRas cells transfected with the indicated siRNAs were treated with TGF-β for 4 days and fixed for FAIRE-seq data acquisition. The data represent the result of two biological replicates. Error bars, standard deviations.

FAIRE-seq analyses and ChIP-seq data using the Etv4 or Etv5 antibodies revealed that matrix metalloproteinase 13 (*Mmp13*), which was included in the identified gene set in Fig. 5F, was a target of Etv4 and Etv5 (Fig. 6A). Knockdown of Etv4/5 resulted in the decreased mRNA expression of Mmp13 (Fig. 6B). We also found that cell invasiveness was inhibited by knockdown of Mmp13 expression (Fig. 6C and Supplementary Fig. S3C).

**Figure 7:**
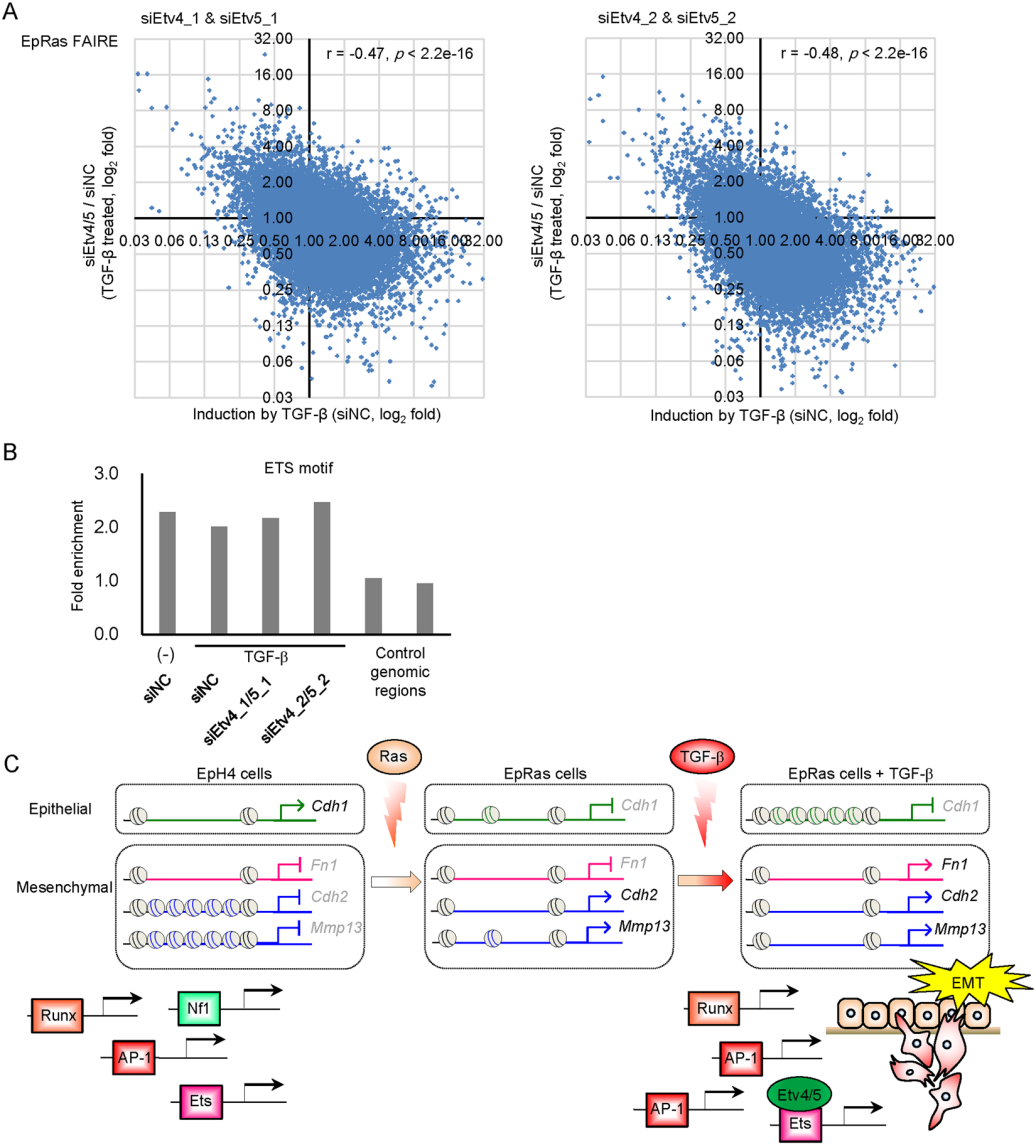
Effect of Etv4/5 siRNAs on the global chromatin accessibility. (**A**) Scatter plots showing the effect of TGF-β (X-axis) and Etv4/5 siRNAs (Y-axis) on the genome-wide chromatin accessibility determined by FAIRE-seq. Note that there was a significant correlation between the effect of two different siRNA sets (r = 0.51, *p* < 2.2e-16, Supplementary Fig. S7). (**B**) Enrichment of ETS binding motif in the FAIRE-positive regions of EpRas cells treated by TGF-β and siRNAs as indicated. (**C**) A schematic model for regulation of EMT-related gene expression through the differential expression of transcription factors and alteration of chromatin accessibility. In the process of EMT, chromatins are gradually closed by Ras and TGF-β at the *Cdh1*/E-cadherin gene locus. In contrast, upregulated genes are regulated by a variety of mechanisms. The *Fn1*/fibronectin gene locus is open irrespective of Ras and TGF-β treatment, while *Cdh2*/N-cadherin gene locus becomes accessible upon Ras transformation, and the *Mmp13* gene locus becomes accessible upon Ras transformation and TGF-β stimulation. Differentially expressed transcription factors, Etv4 and Etv5, are responsible for a subset of EMT-related changes in gene expression partially through the alteration of chromatin accessibility.

Finally, we performed FAIRE-seq analysis after knockdown of Etv4/5 in the presence of TGF-β in EpRas cells. We found that chromatin accessibility at a distal enhancer region, but not at a proximal enhancer region, upstream of the *Mmp13* gene was inhibited by Etv4/5 siRNAs (Fig. 6D). At a genome-wide level, we also found a negative correlation between the effect of TGF-β and Etv4/5 siRNAs on chromatin accessibility (Fig. 7A), while the enrichment of ETS binding motif was not remarkably altered after knockdown of Etv4/5 (Fig. 7B). Taken together, differential expressions of Etv4 and Etv5, members of the oncogenic ETS family, alter chromatin accessibility in TGF-β-stimulated EpRas cells and regulate a subset of EMT-related gene expression associated with the pro-tumorigenic phenotype.

## Discussion

### Altered and/or inducible chromatin accessibility in EMT-prone cells

Recent findings on latent enhancers dynamically regulated by external stimuli in terminally differentiated cells advanced our understanding of transcriptional regulation at the epigenetic level^29, 30^. Importance of transcriptional regulation on protein expression is more appreciated than before, and it was shown that transcriptional regulation contributes to as much as 73% of the protein expression, which is extremely high compared to mRNA degradation (11%), translation (8%), and protein degradation (8%)^31, 32^. Therefore, it is important to evaluate chromatin accessibility, which closely reflects epigenetic status, to reveal mechanisms of gene expression and explain various cellular responses induced by external stimuli.

Our present findings provided direct evidence of the regulatory processes, involving chromatin accessibility during EMT, induced by Ras transformation and TGF-β stimulation. Expression of N-cadherin is regulated at the level of chromatin accessibility, which is determined by Ras transformation. The *Fn1* locus is constantly “open” and is more sensitive to TGF-β signaling than Ras transformation. Finally, chromatin accessibility to *Cdh1* locus is regulated by Ras transformation and TGF-β signaling (after 8-day treatment) (Fig. 7C). Our findings therefore support a previous reports which analyzed individual epigenetic modifications during EMT^25^.

### Roles of DNA binding factors on chromatin accessibility

We found that several motifs were enriched at the accessible chromatin regions in EpH4 and EpRas cells. The AP-1 binding motif was highly enriched and frequently present at the accessible chromatin regions in both cell lines. The AP-1 transcription factors consist of ATF, Jun, and Fos family proteins and play central roles downstream of growth factor signaling. The AP-1 factors, established inducers of EMT, also crosstalk with the Smad signaling pathway at the transcriptional level^33–35^; for example, ATF4 interacts with Sox9, regulates the expression of adhesion molecules of cell-cell junction/cell-extracellular matrix, and induces EMT^36^. Importantly, Biddie *et al*. reported that AP-1 factors increase (or maintain the basal) chromatin accessibility, which enables other transcription factors to bind to the chromatin in the murine mammary epithelial cell line^37^. Our data support their finding, and aberrant Ras signaling may change basal chromatin accessibility and lead to a loss of coordinated regulation of gene expression (with other transcription factors) in EpRas cells (Fig. 7C). The present findings suggest that this is also true for the ETS family transcription factors whose binding motif was enriched both in EpH4 and EpRas cells.

Recently, the cooperative roles of Smads and c-Fos in osteoclastogenesis have been reported. Integrated ChIP-seq and FAIRE-seq analysis revealed that c-Fos and Smads help each other to bind to target genomic regions^38^. In addition, Smad2 and Smad3 bind to TRIM33 to obtain access to and open poised chromatin during differentiation of embryonic stem cells^39^. Based on the established interaction between Smads and AP-1 factors in cancers, it is possible that they are cooperatively involved in the TGF-β-induced changes in chromatin accessibility after EMT induction.

### Regulation of EMT and other pro-tumorigenic phenotypes by oncogenic Etv4 and Etv5

In the present study, we found that Etv4 and possibly Etv5 play a role in the transcriptional control during EMT by TGF-β. Along with Etv1, Etv4 and Etv5 are known as oncogenic ETS family transcription factors. Several chromosomal rearrangements result in overexpression of fusion genes involving Erg, Etv1, and Etv4 in prostate cancer^40, 41^. In a mouse prostate cancer model, Etv4 promotes metastasis following activation of PI3-kinase and Ras signaling^42^, and overexpression of Etv4 induces cell proliferation and EMT in prostate cells^43^. In the mammary gland, expression of Etv4 correlates with expression of ErbB2, but not the estrogen receptor (ER) α in breast cancer^44^. Either dominant-negative Etv4 or knockdown of Etv4/5 inhibits ErbB2-induced tumorigenesis^45, 46^; in addition, Etv4 is required for Wnt1-induced mammary tumorigenesis^47^. Expression of Etv5 also correlates with a poor prognosis of breast cancer patients^48^. A recent report revealed that a transcriptional repressor CIC (also known as Capicua) suppresses invasion and metastasis of mouse lung cancer through inhibition of the expression of Etv4 and its target gene MMP24^49^. Interestingly, MAP kinase inhibits the activity of CIC and induces the expression of Etv1, Etv4, and Etv5, and dysregulated expression of Etv1, Etv4 and Etv5 confer resistance to MAP kinase inhibitors^50^. These findings suggest important roles of Etv4 and Etv5 in both tumorigenesis and tumor progression and metastasis, although some tumor suppressive properties of Etv4 were reported in breast cancer^51, 52^.

The present study suggests that Etv4 and Etv5 play an important role in tumor progression by altering chromatin accessibility and gene expression during EMT. However, the ChIP-seq data shown in the present study suggest that the changes in the chromatin accessibility are not the only mechanism by which Etv4 and Etv5 regulate transcription. Our results also showed that Etv4 and Etv5 regulate only a subset of genes during EMT. These results suggest that EMT may require cooperation with other factors, e.g. other ETS factors, Hmga2, ZEB1 and ZEB2, Snail and Slug, and Twist proteins. Moreover, Etv4 and Etv5 may have pro-tumorigenic functions which are not directly linked to the induction of EMT.

In summary, the alteration of chromatin accessibility by Ras transformation and TGF-β stimulation provides an important mechanism for transcriptional regulation during EMT. The effects of oncogenic Etv4 and Etv5 on these processes in a subset of target genes strengthen our understanding of their roles in breast cancer cells.

## Methods

### Cell culture, antibodies, and reagents

The EpH4 mouse mammary gland epithelial cells and their H-Ras-transformed derivative, EpRas cells, were obtained from Dr. Sabine Maschler (Research Institute of Molecular Pathology, Vienna, Austria)^23, 53^ and cultured in F12/DMEM containing 10% (for EpH4) or 4% (for EpRas) fetal bovine serum (FBS), 50 units/ml penicillin, and 50 µg/ml streptomycin. All cells were maintained in a 5% CO_2_ atmosphere at 37 °C. Of note, EpRas cells express ErbB2 but not ERα and progesterone receptor, according to our RNA-seq data (data available at Gene Expression Omnibus (GEO)). Recombinant TGF-β (TGF-β3) was obtained from R&D systems (Minneapolis, MN, USA). For 8-day cell cultures, medium supplemented with TGF-β was changed every other day and cells were split every 4 days. Mouse monoclonal anti-α-tubulin antibody was purchased from Sigma-Aldrich (St. Louis, MO, USA). Mouse monoclonal anti-E-cadherin antibody was from BD Biosciences (Franklin Lakes, NJ, USA). Rabbit polyclonal anti-Etv4 antibody, previously used for ChIP-seq by Hollenhorst *et al*.^54^, was from AVIVA Systems Biology (ARP32263, San Diego, CA, USA), and mouse monoclonal anti-Etv5 antibody (MAB7107) was from R&D Systems. Anti-phospho-Smad2 antibody (3101) was from Cell Signaling Technologies (Danvers, MA, USA) and anti-Smad2 (ab33875) antibody was from Abcam (Cambridge, UK).

### RNA interference

Transfection of siRNAs was performed according to the protocol recommended for RNAiMAX (Thermo Fisher Scientific, Waltham, MA, USA). We used Stealth siRNAs against mouse Etv4 (siEtv4_1: MSS237548, 5′-AGAAGCUCAGGUACCGGACAGUGAU-3′ and siEtv4_2: MSS237550, 5′-CCUUCUGCAGCAAAUCUCCCGGAAA-3′) and mouse Etv5 (siEtv5_1: MSS272298, 5′-CCCGUUCCUGAAGGCAGAAUCCGAA-3′ and siEtv5_2: 5′-UUAAAUUCCAUGCCUCGGCCAGUCC-3′). The Stealth siRNA sequences were predesigned at BLOCK-iT RNAi Express (Thermo Fisher Scientific) except for Etv5_2 which was designed using BLOCK-iT RNAi Designer. Control Stealth siRNA was purchased from Thermo Fisher Scientific (Cat. 12935-112, sequence not available). siRNAs against mouse Mmp13 (siMmp13_1: 5′-GUAAGUUAUCUUUGAGCAUAC-3′ and siMmp13_2: 5′-CACGUUAACGGACAACUUUCC-3′) (designed using siDirect) and predesigned control siRNA were from RNAi Inc. (Tokyo, Japan). The final concentration of siRNAs in the culture medium was 30 nM.

### RNA-seq

Total RNA was purified from EpH4, EpRas, and siRNA-transfected EpRas cells stimulated with 1 ng/ml TGF-β or left untreated for 48 h. mRNA was purified using the Dynabeads mRNA DIRECT Purification Kit (Thermo Fisher Scientific). Libraries were made using the Ion Total RNA-Seq Kit v2 and directionally sequenced with the Ion Proton using the Ion PI chip v2 and Ion PI IC 200 Kit (Thermo Fisher Scientific), as previously described^55, 56^. Sequenced reads were aligned against the mouse reference transcriptome data (mm10) using TopHat2. Differential gene expression was evaluated using the Cuffdiff function of Cufflink.

### Immunoblotting

Transfected EpRas cells were stimulated with 1 ng/ml TGF-β for 4 or 8 days. Immunoblotting was performed as previously described^57^. Uncropped images are presented in Supplementary Figs S4, S5 and S6.

### FAIRE

The FAIRE analyses were performed based on a previously described protocol^58, 59^. In brief, cells were fixed with 1% (final) formaldehyde for 5 min at room temperature. Fixation was stopped by adding 2.5 M glycine (final 125 mM) for 5 min at room temperature. After washing fixed cells with cold phosphate-buffered saline (PBS) twice, cells were scraped with 1 ml cold PBS and centrifuged. Collected cells were lysed in 800 μl of MC lysis buffer (10 mM NaCl, 10 mM Tris-HCl pH 7.5, 3 mM MgCl_2_, 0.5% NP-40) and incubated on ice for 10 min. After centrifugation at 8,000 rpm at 4 °C for 4 min, the pellet of cells was re-suspended in 400 μl of sodium dodecyl sulfate (SDS) lysis buffer (1% SDS, 50 mM Tris-HCl pH 8.0, 10 mM EDTA, protease inhibitor [P8304; Sigma-Aldrich]) and incubated on ice for 10 min. Next, the cell lysates (containing the DNA) were sonicated for 30 sec for 15 cycles with 30 sec intervals between cycles, using the Bioruptor UCW-201 (Cosmo Bio, Tokyo, Japan). After centrifugation at 14,000 rpm at 4 °C for 10 min, we added 200 μl of ChIP dilution buffer (20 mM Tris-HCl pH 8.0, 150 mM NaCl, 2 mM EDTA, 1% TritonX-100) containing complete EDTA-free protease inhibitors (Roche Diagnostics, Basel, Switzerland). Samples were centrifuged at 8,000 rpm at 4 °C for 4 min. After removal of a control aliquot, supernatants were purified by 3 cycles of phenol/chloroform extraction and reverse-crosslinked by overnight incubation at 65 °C. Genomic DNA was purified by PCR purification kit (Qiagen, Valencia, CA, USA). The FAIRE products were separated by electrophoresis in agarose gels to confirm that the sizes of genomic DNA fragments were approximately 300–400 bp.

### FAIRE-qPCR and FAIRE-seq

Locus-specific primers were designed upstream of the transcription start sites or accessible chromatin regions determined by the FAIRE-seq. Primer sequences for FAIRE-qPCR are shown in Supplementary Table S2. Chromatin accessibility was evaluated by FAIRE-qPCR by calculating an enrichment value of the FAIRE-positive regions of interest relative to the quantities of DNA in FAIRE samples which were determined by Qubit dsDNA HS assay kit (Thermo Fisher Scientific). For FAIRE-seq, total DNA was purified from EpH4 and EpRas cells stimulated with 1 ng/ml TGF-β or left untreated for 8 days. Libraries were made using Ion Plus Fragment Library Kit (Thermo Fisher Scientific) and sequenced with Ion Proton. Sequences were trimmed down to 36 bp and mapped to the murine genome (mm10) using bowtie2. MACS2 (version 2.0.9) was used to identify significantly enriched FAIRE-positive regions at q < 1.0 × 10^−4^. For *de novo* motif prediction, 500 bp DNA sequences flanking the middle position of each FAIRE-positive region were obtained and a Gibbs motif sampler function of CisGenome version 2.0 was used^60^. Promoter regions (within 2 kb of the transcription start sites) and repeat sequences were excluded from the calculation for efficient identification of enriched motifs from the context-specific FAIRE-positive regions^59, 61^. Identified motifs were then evaluated with TOMTOM^62^. Motif enrichment values were calculated using CisGenome by comparison with the matched random genomic sequences. FAIRE-seq data were obtained as two biological replicates. For quantification of FAIRE-seq data, the reads per million mapped reads (RPM) values were calculated for each FAIRE regions, as described^55^. RPM values were log_2_-transformed before correlation analysis.

### RT-qPCR

Quantitative RT-PCR (RT-qPCR) analyses were performed using the StepOnePlus Real-Time PCR System (Thermo Fisher Scientific) and FastStart Universal SYBR Green Master Mix with ROX (Roche Diagnostics). Values of expression were normalized to the mouse TATA binding protein (*Tbp*). The primer sequences are available in Supplementary Table S3.

### Phalloidin staining

Phallodin staining was performed using fluorescein isothiocyanate (FITC)-labeled phalloidin (Sigma-Aldrich) as described^57^.

### ChIP-seq

EpRas cells were cultured in 10-cm plates, treated with 1 ng/ml TGF-β for 1.5 h, and fixed. ChIP was performed as described using 12 μg of antibody per sample^33^. Library construction and ChIP-seq were performed as in FAIRE-seq. Fifteen cycles of PCR amplification was performed before size selection (350–450 bp) by E-Gel SizeSelect (Thermo Fisher Scientific). One-sample analysis of CisGenome^60^ was used for peak calling (sliding window length: 300 bp, p < 0.1).

### Gene ontology analysis

Gene Set Enrichment Analysis (GSEA) was performed as described^63^.

### Matrigel invasion assay

Matrigel invasion assay was performed as described previously^64^. Each experiment was performed as biological triplicates. Migrated cells were stained with crystal violet and quantified using ImageJ based on four field images that were selected randomly for each sample.

### Statistical analysis

The Tukey-Kramer test of the R program (http://www.r-project.org/) was used for multiple comparisons of the data. F-test was performed for the equality of variances. Pearson's correlation test was used for quantitative comparison of FAIRE-seq data.

## Electronic supplementary material

Table S1

Table S2, S3, and Figure S1-7
