# Supplementary material for: Dynamics of chromatin accessibility during TGF-β-induced EMT of Ras-transformed mammary gland epithelial cells
Source: Sci Rep. 2017 Apr 26;7:1166. doi: 10.1038/s41598-017-00973-4 (PMC5430828; doi:10.1038/s41598-017-00973-4)
Supplement: Supplementary file 2 — Table S2, S3, and Figure S1-7 [file 41598_2017_973_MOESM2_ESM.pdf]

Dynamics of chromatin accessibility during TGF-β-induced EMT of Ras-transformed mammary gland epithelial cells

Mayu Arase[1, +], Yusuke Tamura[1, +], Natsumi Kawasaki[1, +], Kazunobu Isogaya[1], Ryo Nakaki[2], Anna Mizutani[1], Shuichi Tsutsumi[2], Hiroyuki Aburatani[2], Kohei Miyazono[1, *], and Daizo Koinuma[1]

[1]Department of Molecular Pathology, Graduate School of Medicine, The University of Tokyo, Bunkyo-ku, Tokyo 113-0033, Japan.

[2]Genome Science Division, Research Center for Advanced Science and Technology (RCAST), The University of Tokyo, Meguro-ku, Tokyo 153-8904, Japan.

[*]Corresponding author.

[+]These authors contributed equally to this work.

SUPPLEMENTARY FIGURE LEGENDS

Supplementary Fig. S1. Expressions levels of *Cdh2, Fn1, Cdh1* and *Esrp2* mRNAs relative to TATA box binding protein (*Tbp*) in EpH4 and EpRas cells. Cells were stimulated with TGF-β for 8 days and harvested for RT-qPCR analysis. Error bars represent standard deviations of the two technical replicates. *: $p < 0.05$.

Supplementary Fig. S2. Expression of AP-1, NF1, CEBP, RUNX, and ETS family genes in EpH4 and EpRas cells. EpH4 and EpRas cells were treated with TGF-β for 48 h and RNA-seq was performed. (A-B) Expression of AP-1 (A), NF1, CEBP, and RUNX (B) family genes. (C) Expression of ETS family genes. Data for Etv4 and Etv5 are shown in Fig. 4A. Error bars, S.D. Horizontal bars indicate statistical significance of the difference at $p < 0.05$.

Supplementary Fig. S3. Motif analysis of the Etv4 and Etv5 binding regions, GSEA analysis of RNA-seq data obtained from TGF-β-treated EpRas cells, and knockdown efficiency of siRNAs. (A) Frequency of the ETS binding motif in the Etv4 and Etv5 binding regions was calculated by MEME-ChIP and CentriMo [65,66]. (B) GSEA analysis was performed as in Fig. 5E and F, using the RNA-seq data obtained from the TGF-β-treated samples. (C) Knockdown efficiency of Mmp13 siRNAs used in Fig. 6C. EpRas cells were transfected with the siRNAs as indicated, and treated with TGF-β for 48 h. Error bars, S.D. of the two technical replicates. siNC: control siRNA, siMmp13_1 and siMmp13_2: two different siRNAs for Mmp13.

Supplementary Fig. S4. Uncropped images of the immunoblotting data in Fig. 4B. Right panels show molecular weight of each marker (kDa, P7706, New England Biolabs, Ipswich, MA, USA).

Supplementary Fig. S5. Uncropped images of the immunoblotting data in Fig. 5A. Right panels show molecular weight of each marker (kDa, P7708, New England Biolabs).

Supplementary Fig. S6. Uncropped images of the immunoblotting data in Fig. 5B. Right panels show molecular weight of each marker (kDa, P7708, New England Biolabs).

Supplementary Fig. S7. Correlation between the effects of two different Etv4/5 siRNAs on chromatin accessibility. A scatter plot showing the effect of siEtv4_1 and siEtv5_1 (X-axis) and siEtv4_2 and siEtv5_2 (Y-axis) on the genome-wide chromatin accessibility determined by FAIRE-seq.

SUPPLEMENTARY TABLES

Supplementary Table S2. Primer sequences for FAIRE-qPCR.

gene			5'→3' sequences
mouse	*Cdh2* (N-cadherin)	Fwd	CCCCAGCTCCTTGATCTCCCGT
		Rev	ACAAATAGCGGGCCTCGGAGT
mouse	*Fn1* (fibronectin)	Fwd	GTGAACAAGTTGTCCTGAGAGG
		Rev	GAAACACCCATTCCACTTGGG
mouse	*Cdh1* (E-cadherin)	Fwd	GCGCACTACTGAGTTCCCAA
		Rev	GACGCCGAGCAAACACTGAG
mouse	*Esrp2*	Fwd	GTCTCTGCAGCTCCCACTAC
		Rev	CAGCAGACAGACGAGCTTGG
mouse	*Gapdh*	Fwd	AGGGCTGCAGTCCGTATTTA
		Rev	AGCTACTCGCGGCTTTACG

Fwd, Forward; Rev, Reverse.

Supplementary Table S3. Primer sequences for RT-qPCR.

gene			5'→3' sequences
mouse	*Mmp13*	Fwd	AGGCCTTCAGAAAAGCCTTCA
		Rev	ATCATGATGTCAGCAGTGCCA
mouse	*Tbp*	Fwd	GCGATTTGCTGCAGTCATCA
		Rev	GCTGCTAGTCTGGATTGTTCTTCA
mouse	*Cdh1*	Fwd	GCTGCTGACCTTCAAGGTGAA
		Rev	TTGACCCTGATACGTGCTTGG
mouse	*Cdh2*	Fwd	TTCCTTGCTTCTGACAATGG
		Rev	ATGTCATAATCAAGTGCTG
mouse	*Fn1*	Fwd	GACAGGAGGAAATAGCCC
		Rev	CATCGTGCAAGGCAACCAC
mouse	*Esrp2*	Fwd	GGACTAGAAACAGATGCTACCGAAGA
		Rev	CTTCGAGAACAACTGACCATTGG

Fwd, Forward; Rev, Reverse.

SUPPLEMENTARY REFERENCES

65 Machanick, P. & Bailey, T. L. MEME-ChIP: motif analysis of large DNA datasets. *Bioinformatics* **27**, 1696-1697 (2011).

66 Bailey, T. L. & Machanick, P. Inferring direct DNA binding from ChIP-seq. *Nucleic Acids Res* **40**, e128 (2012).

Figure S1

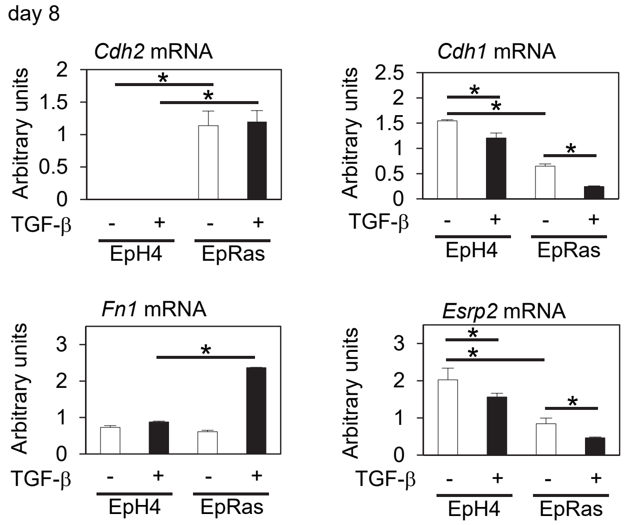
day 8
Cdh2 mRNA
Arbitrary units
2
1.5
1
0.5
0
*
*
TGF-β
-
+
-
+
EpH4
EpRas
Cdh1 mRNA
Arbitrary units
2.5
2
1.5
1
0.5
0
*
*
*
Fn1 mRNA
3
2
1
0
*
Esrp2 mRNA
3
2
1
0
*
*
*

Figure S2

A B C

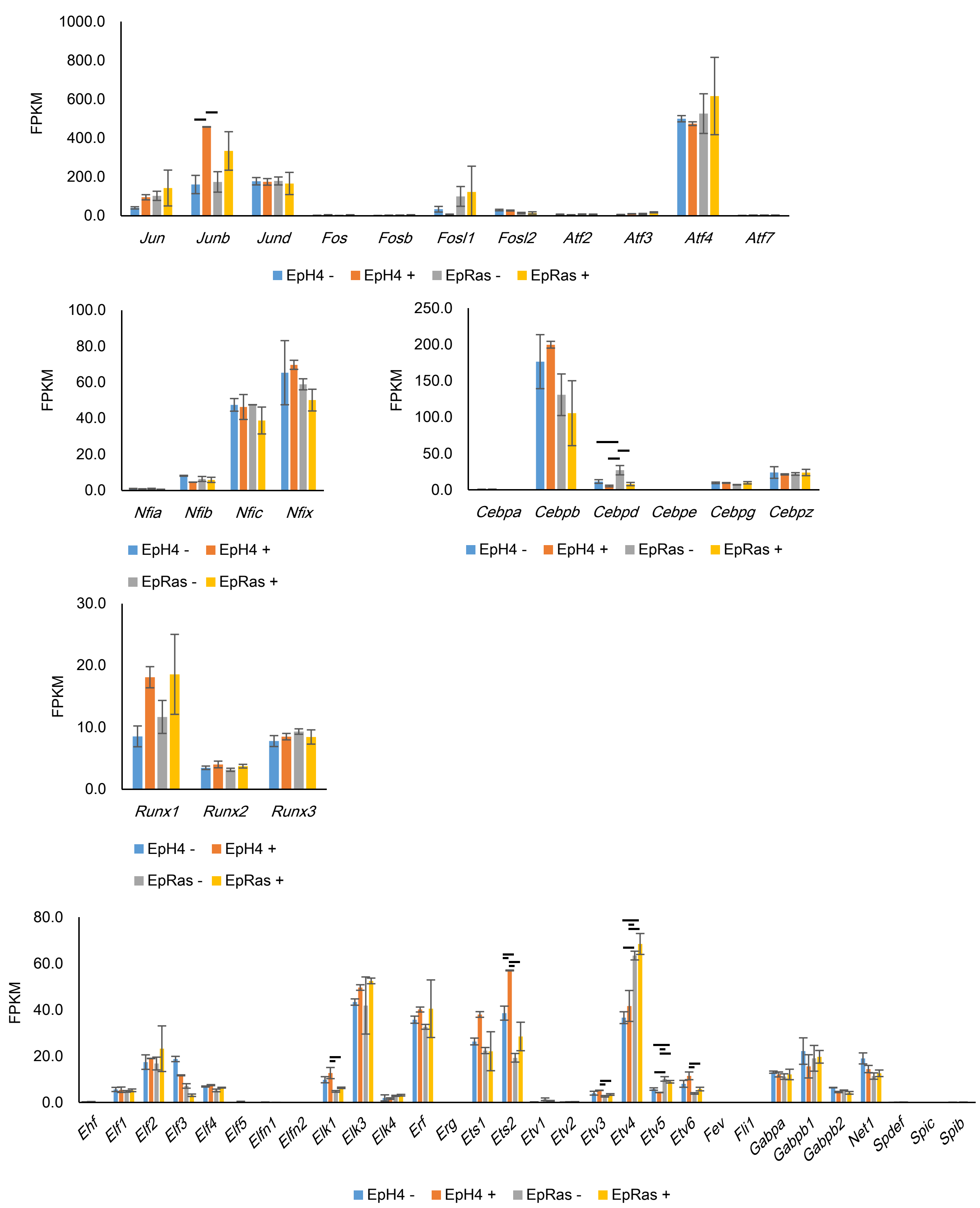

Figure S3

A

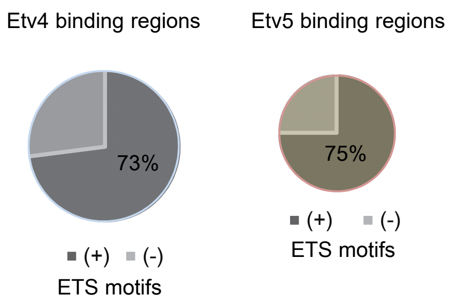

B

	SIZE	NES	p-value	FDR q-value
ACTIN_FILAMENT_BINDING	17	1.72	0.0000	0.075
LOCOMOTORY_BEHAVIOR	19	1.71	0.0000	0.050
CELL_MIGRATION	37	1.63	0.0000	0.157
BEHAVIOR	26	1.62	0.0010	0.141
EPIDERMIS_DEVELOPMENT	29	1.60	0.0000	0.153
EXTRACELLULAR_REGION	111	1.59	0.0000	0.132
EXTRACELLULAR_REGION_PART	83	1.57	0.0000	0.170
PROTEINACEOUS_EXTRACELLULAR_MATRIX	31	1.55	0.0000	0.184
TISSUE_DEVELOPMENT	50	1.54	0.0000	0.186
ACTIN_BINDING	46	1.54	0.0000	0.187

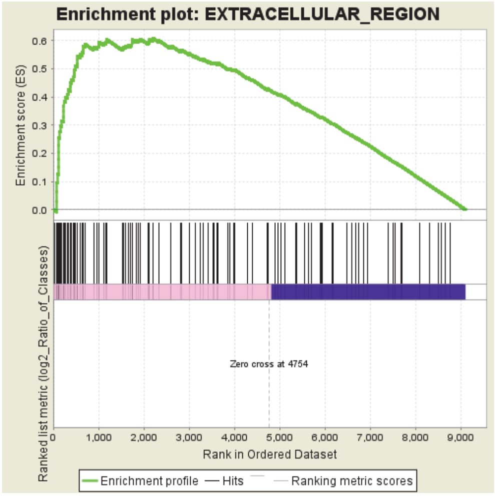

C

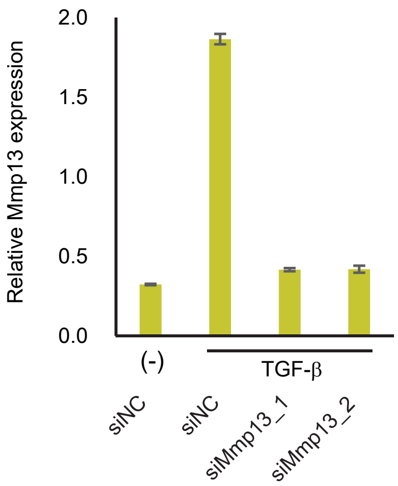

Figure S4

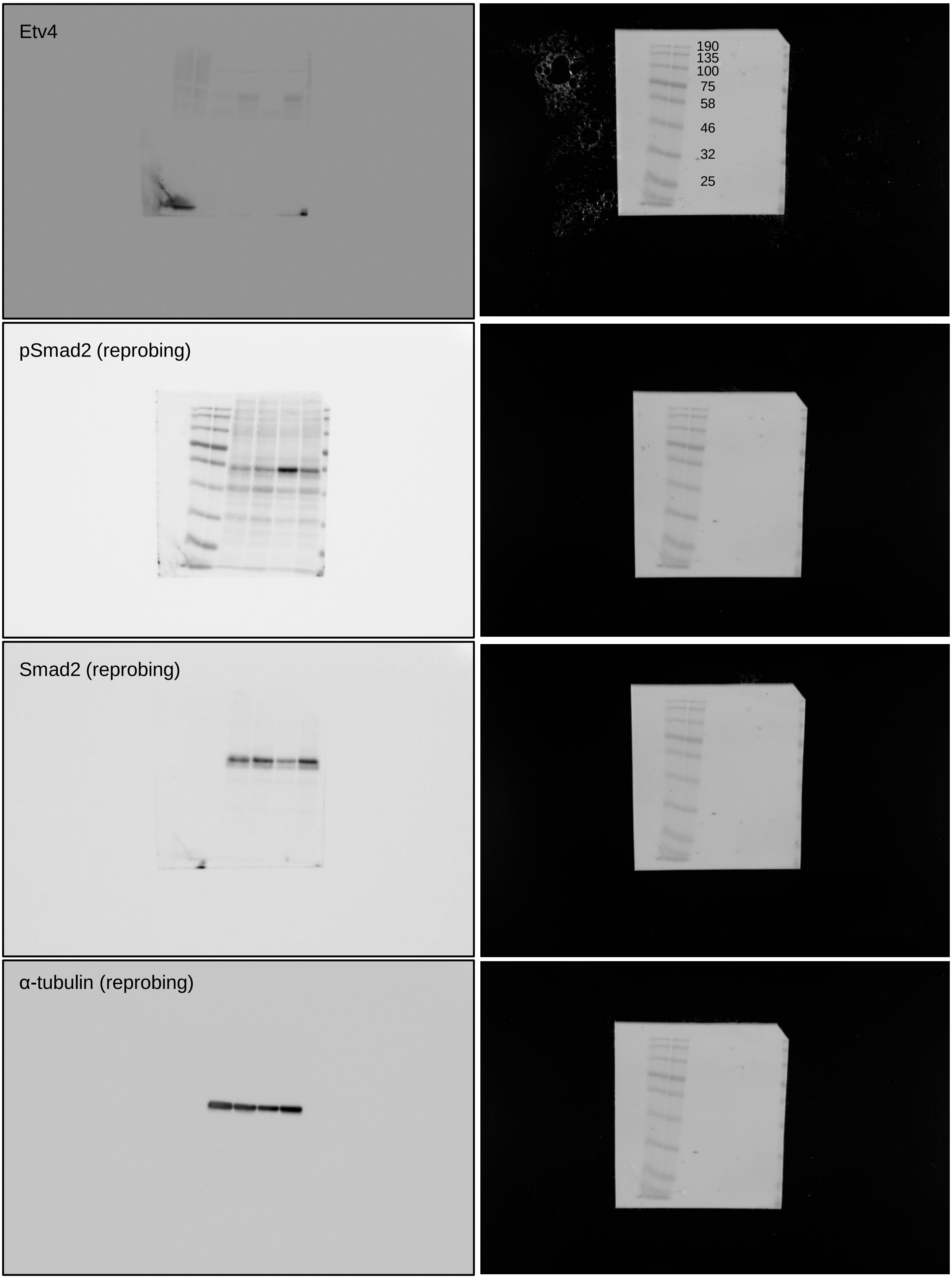
Etv4
190
135
100
75
58
46
32
25
pSmad2 (reprobing)
Smad2 (reprobing)
α-tubulin (reprobing)

Figure S5

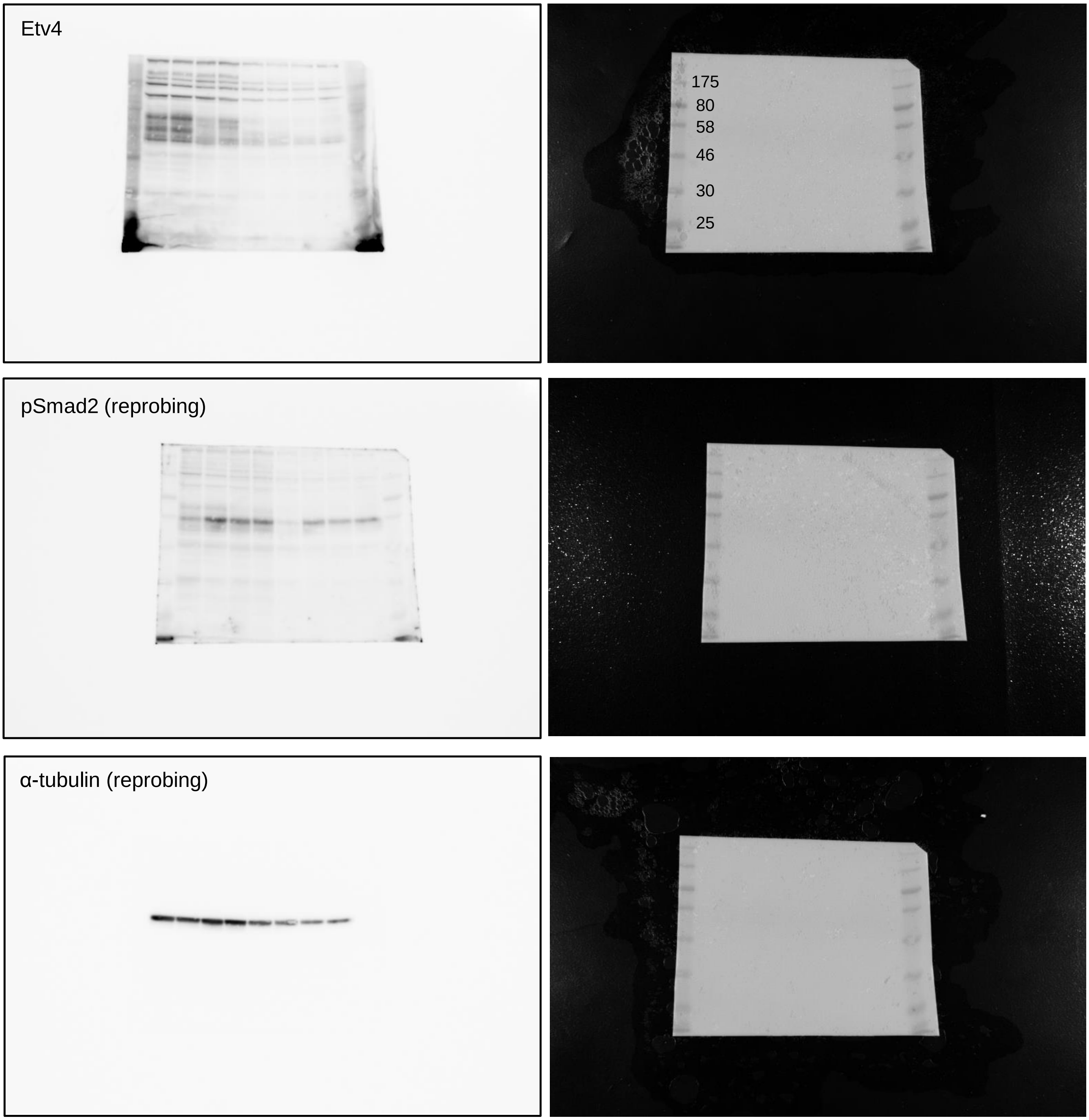

Figure S6

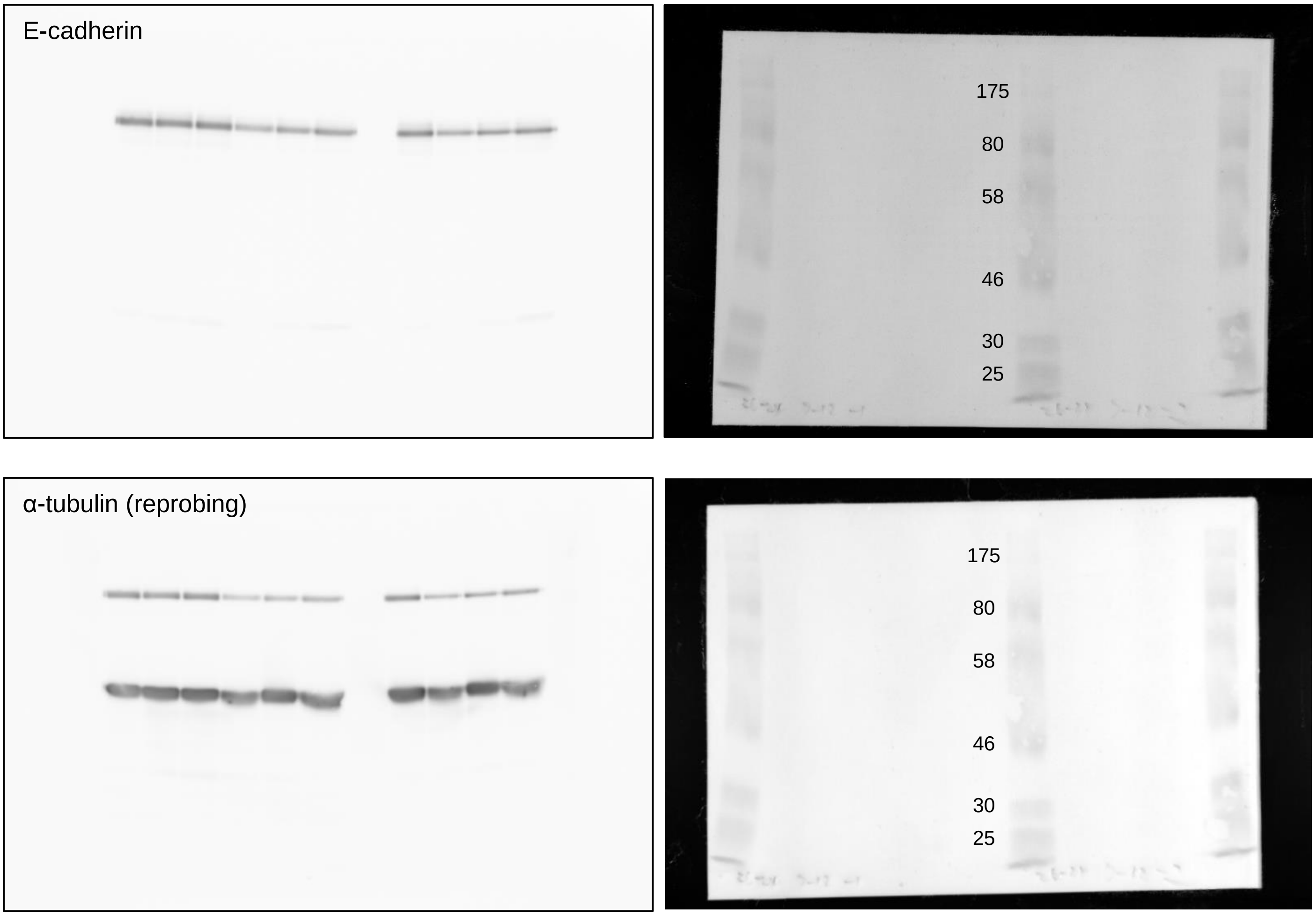
E-cadherin
175
80
58
46
30
25
α-tubulin (reprobing)
175
80
58
46
30
25

Figure S7

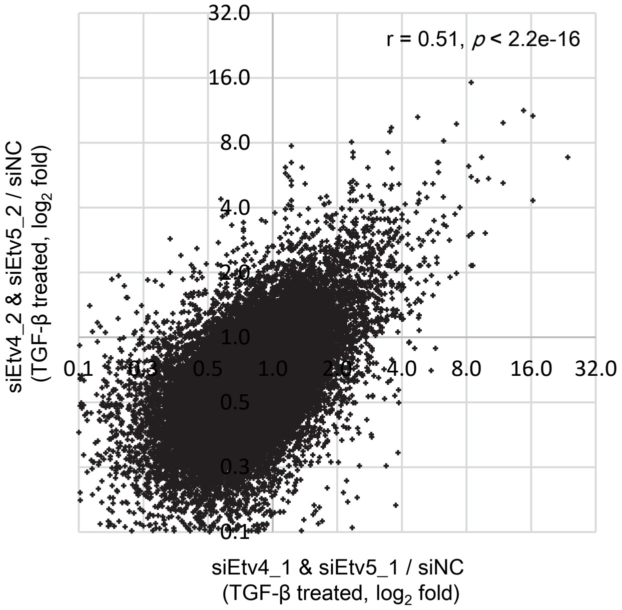
r = 0.51, p < 2.2e-16
32.0
16.0
8.0
4.0
2.0
1.0
0.5
0.3
0.1
0.1
0.3
0.5
1.0
2.0
4.0
8.0
16.0
32.0
siEtv4_2 & siEtv5_2 / siNC
(TGF-β treated, log2 fold)
siEtv4_1 & siEtv5_1 / siNC
(TGF-β treated, log2 fold)
